# Special considerations in paediatric burn patients

**DOI:** 10.4103/0970-0358.70719

**Published:** 2010-09

**Authors:** Ramesh Kumar Sharma, Atul Parashar

**Affiliations:** Department of Plastic Surgery, Postgraduate Institute of Medical Education and Research, Chandigarh – 160 012, India

**Keywords:** Burn injuries, paediatric, special considerations

## Abstract

Burn injuries are a major cause of morbidity and mortality in children. In India, the figure constitutes about one-fourth of the total burn accidents. The management of paediatric burns can be a major challenge for the treating unit. One has to keep in mind that “children are not merely small adults”; there are certain features in this age group that warrant special attention. The peculiarities in the physiology of fluid and electrolyte handling, the uniqueness of the energy requirement and the differences in the various body proportions in children dictate that the paediatric burn management should be taken with a different perspective than for adults. This review article would deal with the special situations that need to be addressed while treating this special class of thermal injuries. We must ensure that not only the children survive the initial injury, but also the morbidity and complications are minimized. If special care is taken during the initial management of paediatric burn injuries, these children can be effectively integrated into the society as very useful and productive members.

## INTRODUCTION

Burn-related injuries are a leading cause of morbidity and mortality in children. Burn injuries rank third among injury-related deaths in children aged 1 to 9 years.[1] In India, paediatric burns account for 17–25% of total burn admissions.[2,3] Approximately 90% of burns are caused by household accidents. In children younger than three years, scalds are responsible for most of the burns.[4] Scald burns usually occur when a child accidentally pulls the container with hot liquid onto himself. It may also result from bathtub submersion injuries usually by an unattended child. In older children, flame burns are more common. Firecracker injuries and household fires are the common aetiologic factors for these burns, which are often of full thickness.

Management of the children with major burns taxes skills of the personnel of any unit. Appreciating the major differences between burn management in children and adults is important. Children have nearly three times the body surface area (BSA) to body mass ratio of adults. Fluid losses are proportionately higher in children than in adults. Consequently, children have relatively greater fluid resuscitation requirements and more evaporative water loss than adults. The large BSA to body mass ratio of the child also predisposes the child to hypothermia, which must be aggressively avoided. Children younger than two years have thinner layers of skin and insulating subcutaneous tissue than older children and adults. As a result, they lose more heat and water than adults do, and they lose these more rapidly than adults. In very young children, temperature regulation is partially based on non-shivering thermogenesis, which further increases metabolic rate, oxygen consumption, and lactate production. In addition, because of disproportionately thin skin, a burn that may initially appear to be of partial thickness in a child may instead be of full thickness in depth. Thus, the child’s thin skin may make initial burn depth assessment difficult.

## INITIAL TREATMENT CONSIDERATIONS IN PAEDIATRIC BURNS

Rapid assessment and treatment of immediate life-threatening conditions is mandatory in patients with burns. Endotracheal intubation is indicated in children with respiratory distress or airway compromise caused by airway oedema. Because of the small diameter of the paediatric airway, a low threshold for intubation should be maintained. Children with burns affecting more than 10% of the BSA should receive intravenous fluid resuscitation. Burn wounds should initially be covered with dry sterile sheets, and a thorough history and physical examination should be obtained. Patients should be kept warm by infusing warm intravenous fluids, elevating room temperatures, and minimizing patient exposure. Tetanus immunization should be administered as indicated.

### Admission criteria

Hospital admission criteria for paediatric patients with thermal injury include the following:

- Partial thickness burns greater than 10% of total BSA (TBSA)
- Full thickness burns greater than 2% of TBSA
- Burns involving the face, hands, genitalia, perineum, or major joints
- Circumferential extremity burns
- All high-voltage electrical burns, including lightning injury
- Admission of low-voltage electrical burns is selective
- Chemical burns
- Inhalation injury
- Burn injuries in patients with pre-existing medical disorders that could complicate management, prolong recovery, or affect mortality (e.g. diabetes, immunosuppression)
- Suspected child abuse
- Cases in which it is determined that it is in the best interest to admit the child (i.e. parental inability to care for the burn)

### Inhalation injury

Clues to inhalation injury include increased respiratory rate, hoarseness, being burned in an enclosed space, altered mental status, head and neck burns, singed nasal hairs, inflamed oral mucosa, and carbonaceous sputum. Indications for intubation include compromised upper airway patency, the need for ventilatory support as manifested by poor gas exchange or increased work of breathing, or compromised mental status. Correlation of the history and clinical findings comprise the most practical approach to determining the need for intubation. In children, the larynx is more cephalad and they are also likely to deteriorate faster than adults in terms of upper airway oedema and alveolar-capillary block. Further, repeated intubation attempts may cause oedema and obstruction. For these important reasons, experience in paediatric intubation is needed. Once an airway is established, securing the airway well is important, especially in patients with facial burns, to avoid accidental extubation. This is usually achieved by fixing the endotracheal tube with umbilical tape or other fabric tape tie.

### Burn depth

The depth of burn is classified as follows:

#### Superficial partial thickness

These burns are superficial with injury to the epidermis and superficial dermis. These are second-degree burns and are characterized by ruptured weeping blisters. They are also erythematous and painful. Superficial partial-thickness burns spontaneously heal within two weeks, usually without scarring.

##### Deep partial thickness

These are deep burns with injury to the epidermis and deeper dermis, but some viable dermis remains. These are also considered second-degree burns but are whiter and less erythematous as the depth into the dermis increases. Distinguishing between deep partial-thickness burns and full-thickness burns may initially be difficult. Deep partial-thickness burns heal spontaneously but often after 3–4 weeks. The degree of scarring is related to the length of time needed for re-epithelialization.

##### Full thickness

In these cases, injury to the epidermis and entire dermis occurs. These are third-degree burns that typically are white, brown, or black. The eschar is leathery and insensate. These burns do not heal spontaneously (except for very small ones that heal by wound contraction).

##### Burn size

**Figure 1:**
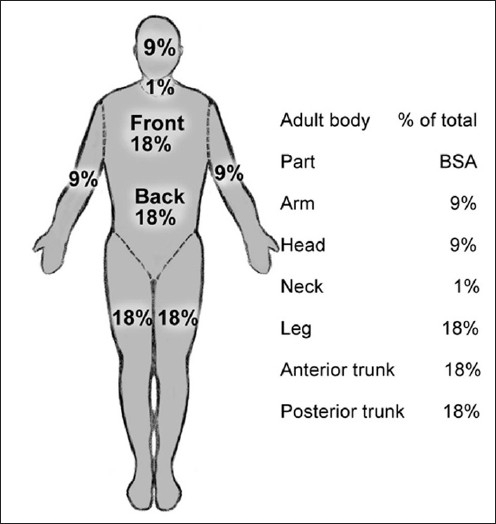
“Rule of Nines” as in adults

In children, the relative BSA of the head and neck is much larger than in adults, and the BSA associated with lower extremity is much less. The Rule of Nines [Figure 1], devised by Pulaski and Tennison, is a useful and practical guide for calculating the extent of the burn in adult patients, but some modifications need to be made while applying this formula to the children. The adjustment of the percentage of BSA in children according to age is depicted in Table 1. This remains a rapid method for initial assessment of paediatric burns.

**Table 1:** Percent of body surface area according to age

	*New born*	*3 years*	*6 years*	*12±years*
Head	18%	15%	12%	6%
Trunk	40%	40%	40%	38%
Arms	16%	16%	16%	19%
Legs	26%	29%	32%	36%

**Figure 2:**
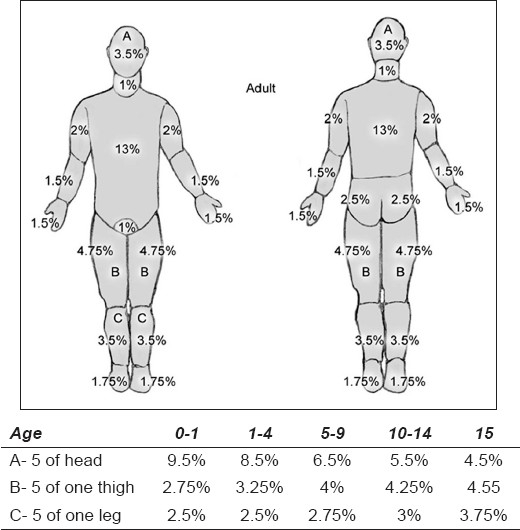
Lund and Browder chart (with age appropriate measurements of BSA)

However, Lund and Browder[5] charts can also be used to more precisely calculate the percentage of BSA burned by mapping the injured areas of the body on charts detailing age-appropriate measurements [Figure 2].

#### Electrical injuries

Electrical burns require special consideration. Low-voltage injuries result from sources of less than 1000 V and include oral injuries from biting electrical cords, outlet injuries from placing objects into wall sockets, and injuries from contacting live wires or indoor appliances. High-voltage injuries are caused by sources of more than 1000 V and result from contact with a live wire outdoors or from being struck by lightning. Children who have sustained high-voltage electrical injury require admission to the hospital with cardiac monitoring, serial electrocardiography, urinalysis and determination of creatine kinase and urine myoglobin levels. Creatine kinase levels rise as a result of massive tissue destruction especially of muscle. Chromoproteins like myoglobin can precipitate and cause renal failure. Thus, myoglobinuria and haemoglobinuria should be aggressively treated with hydration, osmotic diuretics, and alkalinization of the urine. Osmotic diuretic like mannitol is useful to maintain a urine output of 1 to 2 ml/kg/hour. Alkalinizing the blood helps in increasing the solubility of chromoproteins in the urine, thus preventing its precipitation in the tubules. This requires addition of bicarbonate to the resuscitation solution. After the pigments have cleared, the urine flow is titrated at 1 ml/kg/hour. In addition, extremities must be carefully monitored for the development of compartment syndrome, necessitating escharotomy or fasciotomy. Appropriate radiographic examinations should be performed to exclude concomitant long bone injury. Many children who have sustained low-voltage electrical injury can be treated as outpatients as long as they have no history of loss of consciousness and no cardiac dysfunction during 4–6 h observation in the hospital.

#### Chemical burns

Chemical burns in children are treated as in adults. Saturated clothing should be removed, powdered chemicals should be brushed off the skin, and the contaminated area irrigated with copious amounts of water for at least 20 minutes and until the patient experiences a decrease in pain in the wound. There has been considerable discussion about the use of neutralizing solutions in treatment of chemical burns. However, control of amount of strong neutralizing solution is the key difficulty. In addition, there is risk of exothermic reaction with concentrated solutions. These solutions are useful when they are of buffered variety and are correctly applied after copious irrigation with water. Chemical injuries to the eye are treated by forcing the eyelid open and flushing the eye with water or saline. With gasoline injuries, the petroleum products may cause severe full-thickness cutaneous tissue damage, and absorption of the hydrocarbon may cause pulmonary, hepatic, or renal failure.

## FLUID RESSUSCITATION

The importance of appropriate resuscitation cannot be overemphasized. An inadequate fluid resuscitation results in tissue ischaemia, renal failure, and extension of indeterminate depth wounds to full-thickness injury. Overzealous fluid administration results in pulmonary oedema, heart failure, compartment syndrome (either of the extremities, chest, or the abdomen), and extension of indeterminate wounds to full-thickness injury.[6]

In small burns, parenteral fluid resuscitation is not needed. As a rule of thumb, a child with less than 10% BSA superficial burns does not need parenteral fluids. However, in the presence of deep burns parenteral resuscitation may be needed. Thus, each case needs to be individualized. An infant refusing anything orally may necessitate parenteral fluids.

Fluid resuscitation requires knowledge of the size of body fluid compartment and water metabolism in a burned child. In infants, approximately 50% of the body weight is extracellular. This decreases to 35% in a young child and 20% in adult. The higher metabolic rate of infants is responsible for higher insensible and renal water losses than in adult. In addition, the ability of the immature kidney to excrete concentrated urine above 800 mmol/l is limited. Therefore the ability to conserve water is limited and dehydration can develop very rapidly. Thermal injury produces major fluid losses from a variety of causes within first 24–36 hours. Immediately after thermal injury, there is increase in capillary permeability. Water, electrolytes and proteins freely escape from the intravascular to the interstitial spaces. Generalized oedema ensues with predominance in the area of injury. Vascular damage is thought to be due to both direct heat injury and due to vasoactive substances released from burned tissue. Fluid loss has been documented to be most severe during the eight hours immediately after the burns. Therefore most resuscitation formulae suggest giving half of the 24 hours requirement in first eight hours.

### Fluid formulae

The children have a limited physiologic reserve. Thus, fluid resuscitation in a child needs to be more precise than that for an adult with similar burn. Children also require more fluid for burn shock resuscitation than adults with similar thermal injury.[7,8] Weight-based formulas tend to under-resuscitate small injuries in small children, sometimes providing even less than maintenance fluid requirements, and grossly over-resuscitate large injuries in older children.[9] Therefore, a more appropriate means of calculating fluid resuscitation requirement is based on BSA burned. Such a formula has been developed as follows:

#### Total requirement for first 24 hours

$$2000\;\mathrm{ml}\;/m^{2}\;\mathrm{BSA}\;+\;5000\;\mathrm{ml}\;/m^{2}\;\mathrm{BSAB}\;\mathrm{(Body}\;\mathrm{Surface}\;\mathrm{Area}\;\mathrm{Burned)}.\;$$

Half of the total fluid allowance is administered during first eight hours, the other half during the next 16 hours. The fluid generally used is Ringer lactate.

Warden[10] from Shriners Burn Institute modified the Parkland Formula for children with addition of maintenance fluid requirement. He reported adequate resuscitation with this modified Parkland formula:

$$\mathrm{Total}\;\mathrm{requirement}\;\mathrm{for}\;\mathrm{first}\;24\;\mathrm{hours}\;=\;4\;\mathrm{ml}/\mathrm{kg}/\%\mathrm{TBSA}\;+\;1500\;\mathrm{ml}/m^{2}\;\mathrm{BSA}$$

Because paediatric burn victims are also more prone to hypothermia caused by the loss of integument, initial fluid should be warmed. If a patient is presented after some period of delay and has not been resuscitated properly during that time, adjustments should be made in the calculated fluid requirements to take these factors into account. Infants are at risk of developing hypoglycaemia because of limited glycogen stores; therefore, glucose levels should be monitored, and Ringer lactate solution with 5% dextrose should be used for maintenance fluids. Factors that may increase fluid requirements include delayed resuscitation, associated trauma, inhalation injury, pre-injury dehydration and the need for escharotomy, fasciotomy, or both.

Evaluation of adequacy of resuscitation in children is difficult. Clinical signs of hypovolaemia like low-blood pressure and decreased urine output may not present until more than 25% of circulating volume is lost and complete cardiovascular decompensation is imminent. Adequacy of fluid resuscitation can be assessed by monitoring sensorium, and peripheral circulation and arterial blood gas analysis are helpful to assess the resuscitation. Urine output is one of the most sensitive indicator and it should be maintained at 1 ml/kg/hour.

Children may develop compartment syndrome requiring fasciotomy of non-burned extremities after the administration of large amounts of intravenous fluid. Cerebral oedema may also occur with massive resuscitation, and care should be taken to maintain cerebral elevation during the administration of large volumes of fluid to a child, particularly during initial 24–48 hours post-burn. Abdominal compartment syndrome, which compromises renal perfusion, pulmonary function, and cardiac output, can occur because of massive administration of fluid during the resuscitative period.[11] A nasogastric tube should be placed in all children with burns more than 20% TBSA or in intubated children with burn injury. Abdominal distension caused by swallowed air from crying and intubation are extremely common in children, and the nasogastric tube commonly will decompress distension because of air in the stomach. Abdominal compartment syndrome, in which the abdomen is distended from fluid, is diagnosed by measuring bladder pressure via the Foley catheter using a standardized technique.[12] Pressures more than 30 mmHg mandate intervention, which usually consists of either placement of an intra-abdominal catheter to evacuate abdominal cavity fluid or abdominal exploration to relieve abdominal pressure.

Failure of infant or child to respond to fluid resuscitation is generally due to inadequate fluid administration because of inaccurate assessment of the BSA burnt. The discrepancy in BSA and body mass also results in rapid loss of body heat to the environment leading to hypothermia and its attendant impaired cardiovascular response to resuscitation.

By the end of 24 hours, most of the burned patients are haemodynamically stable. Although heat-injured micro-vessels continue to manifest increased vascular permeability for several days, the rate of loss is considerably less than that seen in first 24 hours. The patient’s post-resuscitation fluid requirement can be calculated by adding their evaporative losses to their maintenance fluid requirement. This can be calculated by using any of the following formulae in paediatric burns:

$$\begin{array}{l} \mathrm{Daily}\;\mathrm{fluid}\;\mathrm{requirement}\;(\mathrm{Post}\;\mathrm{resuscitation})\;=\;\\ (35\;+\;\%\mathrm{burn}\;\;\mathrm{TBSA})\;\times \;\mathrm{BSA}\;(m^{2})\;\times \;24\;+\;1500\;\mathrm{ml}/m^{2}\;\mathrm{BSA}\;\mathrm{or}\;3750\;\mathrm{ml}\;\mathrm{BSAB}\;(m^{2})\;\mathrm{per}\;\mathrm{day}\;+\;1500\;\mathrm{ml}/m^{2}\;\mathrm{BSA} \end{array}$$

## PAIN MANAGEMENT

Appropriate management of pain in the child with burn injury can be challenging, but is important in maximizing their outcome. Children will often thrash, resist care, and dislodge important treatment adjuncts (such as the endotracheal tube or intravenous catheter) when in pain.[13] Small doses of intravenous narcotics (0.1 mg/kg of morphine) can initially be administered during the resuscitative period to decrease pain. If the patient remains haemodynamically stable and shows no evidence of respiratory depression after this dose, the dose can be increased if the patient continues to experience pain. Intramuscular absorption of narcotics is erratic and potentially dangerous during resuscitation because of delayed absorption and should be avoided. Intravenous paracetamol is a good adjunct along with opioids in the acute pain management. The IV route allows rapid passage of paracetamol in the systemic circulation leading to a rapid onset and faster distribution resulting in higher plasma concentration as compared with oral and rectal route. Meyer and colleagues[14] described the use of paracetamol in the treatment of background pain in children after acute burn injury and found that in 50% of these children, especially the youngest and those with smaller burns, did not require any morphine.

## NUTRITION

Burned children, similar to adults, have a markedly increased caloric requirement because of the development of a hypermetabolic response after burn injury. This hypermetabolic state is caused by the burn itself, catecholamine release after injury, pain and anxiety, operative interventions, and tissue metabolic demands. In general, a child with a burn greater than 20–30% will require placement of a nasoduodenal feeding tube to provide needed caloric supplementation.

Early initiation of enteral feedings can decrease the need for glucose-containing intravenous infusions during resuscitation. Most children will tolerate enteral feeds as early as 3–6 hours post-burn. Early administration of enteral nutrition has been shown to be efficacious in children.[15,16] The child’s caloric requirements can be calculated using modifications of standard formulas, such as the Curreri Junior formula.[17]

### 

#### 

##### Curreri junior formula:[18]

$$\begin{array}{l} 0–1\;\mathrm{year}\;–\;\mathrm{BMR}\;+\;15\;\mathrm{kcal}/\%\mathrm{burn} \end{array}$$

$$\begin{array}{l} 1–3\;\mathrm{year}\;–\;\mathrm{BMR}\;+\;25\;\mathrm{kcal}/\%\mathrm{burn} \end{array}$$

$$\begin{array}{l} 4–15\;\mathrm{year}\;–\;\mathrm{BMR}\;+\;40\;\mathrm{kcal}/\%\mathrm{burn} \end{array}$$

Alternative formula can be SBI – Galveston revised formula (1–11 years);[19]

Daily calorie requirement: 1800 kcal/m^2^ + 1300 kcal/m^2^ BSAB

Matsuda *et al*.[20] have suggested that any patient with more than 10% burns should receive a dietary regimen containing a non-protein calorie to nitrogen ratio of 100:1 in order to achieve a positive nitrogen balance. This co-relates with a protein intake of 20% of total calories. Carbohydrates should provide about 48% of the total calorie intake, the fats providing the rest of the caloric need.[21]

## SYSTEMIC ANTIBIOTICS

Prophylactic systemic antibiotics are not used in the treatment of burn patients because this increases the risk of infection with resistant organisms. Instead, the use of systemic antibiotics is reserved for the treatment of specific infections, with antibiotics administered at the first sign of clinical infection. Antibiotic regimens are then modified as culture results and thus, antimicrobial sensitivity results become available. Burn wound cellulitis refers to infection spreading in dermal lymphatics in the non-burned skin surrounding a burn, usually occurring in the first few days after burn injury. Burn cellulitis is commonly caused by *Streptococcus pyogenes*. Prior to the antibiotic era, Streptococcus pyogenes (group A beta-hemolytic streptococci) was the predominant pathogen implicated in burn wound infections and was a major cause of death in severely burned patients. Staphylococcus aureus became the principal etiological agent of burn wound infections shortly after the introduction of penicillin G in the early 1950s, which resulted in the virtual elimination of Streptococcus pyogenes as a cause of infection in thermally injured patients. Although Staphylococcus aureus remains a common cause of early burn wound infection, Pseudomonas aeruginosa from the patient’s endogenous gastrointestinal flora and/or an environmental source is the most common cause of burn wound infections in many centers[22]. Invasive burn wound sepsis leads to systemic toxicity with high fever, bacteraemia, and a hyperdynamic circulatory state with hypotension and cardiovascular collapse. Diagnosis can be made by either clinical examination, or by quantitative burn wound cultures or burn wound histology.

## MANAGEMENT OF THE BURN WOUND

The ultimate aim of wound management in burns is to prevent wound infection and thereby facilitate closure of wounds either spontaneously as in superficial burns or provide coverage to the raw areas by autogenous skin grafts.

The blisters in a *second-degree superficial* burn should as a rule be kept intact as it is supposed to aid in early epithelialization. However, if they are too big or covering the eyes or orifices, they can be de-roofed. Devitalized skin and ruptured blisters should be debrided. Topical antibiotic therapy should be used to delay bacterial colonization. Silver sulphadiazine cream is a commonly used broad-spectrum topical antimicrobial cream. It is applied as a thin layer with gauze dressings twice a day. Facial burns are usually treated with a combination of antimicrobial product containing polymyxin B, neomycin, and bacitracin (e.g. Neosporin ointment) The use of silver sulphadiazine cream is avoided on the central face because it may cause severe ocular irritation. The thin subcutaneous tissue in the ears predisposes to the development of chondritis. Ear burns should be treated with mafenide cream because of its excellent cartilage penetration properties. Successful burn wound management in children demands conversion of open wounds to close wounds as soon as possible. The concept of early removal of burn eschar and immediate wound closure has gained widespread acceptance. Evidence suggests that early eschar removal is effective in decreasing morbidity and improving the mortality rate.[23,24] Full-thickness burns (with the exception of very small injuries that are allowed to heal by contraction) should be grafted. The goal is to excise the wound within the first week of injury. Additionally, deep partial-thickness burns that take longer than three weeks to heal usually benefit from grafting, with less hypertrophic scarring and better cosmetic result. The excised areas are covered with autograft, or temporarily with some biological dressing, till definitive autograft is available.

Preoperatively, patients must be haemodynamically sound and have optimal acid-base, fluid, and electrolyte balance. Adequate blood must be available before considering excision and grafting. A prophylactic dose of a first-generation cephalosporin antibiotic may be used. Burn wound excision and grafting is generally undertaken around 5–7 days post-burn and 5–15% BSA is excised at a time.

Attention to maintain the body temperature at all times is extremely important. Burn excision involves tangential removal of thin slices of eschar until profuse pinpoint bleeding from a moist, viable, deep dermal surface, or subcutaneous fat is observed. Meticulous haemostasis is then obtained using epinephrine-soaked (1:100,000) sponges, topical spray thrombin, and electrocautery, followed by immediate grafting with thin sheets of autograft. The grafts are then applied to the wound bed and secured.

Autograft skin is obviously preferred whenever possible. Unfortunately, patients with large burns may not have enough autologous skin available for complete coverage. In such patients, burns can be excised and temporarily covered with numerous biologic dressings (e.g. cadaveric skin, pigskin) or skin substitutes. As more donor sites become available, the temporary wound covers are removed and the wounds are grafted.

### Further management

For burns that take longer than three weeks to heal or for wounds that have been grafted, hypertrophic scarring remains an important sequelae. It can be minimized with the use of compression therapy with custom-made garments that apply 25–30 mmHg pressure to such areas. Gel pads can be added underneath or sewn into the garments to apply extra compression. Compression therapy is continued throughout the wound healing process (approximately 12–18 months). Lotion application with massage therapy is used to keep the healed or grafted areas soft and supple. Hypertrophic scar formations over joints result in contractures across the joints with concomitant decreased range of motion. Aggressive attention to physical therapy, with appropriate consultation, is necessary to ensure optimal results. Active and passive range of motion exercises are instituted and splints are worn at night and between exercise periods. Patients with burns are at risk for contractures and should be followed for years to monitor for the development of these complications.

## OUTCOME AND PROGNOSIS

With the exception of infants, the prognosis for survival in children and adolescents is quite good. The most important factor that has lead to improvement in prognosis is the prompt identification, excision, and effective wound closure.[25] Besides, strides have been made in resuscitation, intensive care, antimicrobials, vascular access, nutritional support, and skin banking. However, presence of co-existent inhalational injury places the child at higher risk of mortality.[26] Thus, at the present time most children with large burns in absence of inhalational injury should survive their injuries.

## FUTURE RESEARCH AND CONTROVERSIES

Numerous areas in both the clinical and basic sciences are undergoing active research. One such area of interest is the hypermetabolic response to severe burns and the association with increased energy expenditure and muscle-protein catabolism. Studies have investigated different mechanisms to attenuate the muscle-protein catabolism that occurs frequently, despite appropriate nutritional support, in children with large burns.[27] These studies are promising because attenuation of muscle-protein losses may improve strength and ability to recuperate. A prospective randomized controlled trial of recombinant human growth hormone in combination with the beta-blocker propranolol demonstrated attenuated hypermetabolism and inflammatory and acute phase responses after severe burn injury.[28] Human growth hormone improves post-traumatic hypermetabolism, but its use alone is associated with hyperglycaemia and increased free fatty acids and triglycerides. Concomitant administration of propranolol improved fat metabolism and insulin sensitivity and avoided the adverse effects of recombinant growth hormone alone.

Another active area of research is in the development of cultured skin to treat very large burns. At present, cultured epidermal autografts (CEAs), which are grown from the patient’s own uninjured epidermis, are used. However, these grafts are very thin and fragile. In the future, cultured bilayered skin (epidermis and dermis) should lead to better functional and cosmetic results.
